# Impact of river discharge seasonality change on tidal duration asymmetry in the Yangtze River Estuary

**DOI:** 10.1038/s41598-020-62432-x

**Published:** 2020-04-14

**Authors:** Xiayan Yu, Wei Zhang, A. J. F. Hoitink

**Affiliations:** 1 State Key Laboratory of Hydrology-Water Resources and Hydraulic Engineering, Hohai University, Nanjing, 210098 China; 2 College of Harbor, Coastal and Offshore Engineering, Hohai University, Nanjing, 210098 China; 3 Hydrology and Quantitative Water Management Group, Wageningen University & Research, Droevendaalsesteeg 3, 6708 Wageningen, The Netherlands

**Keywords:** Hydrology, Engineering

## Abstract

The Yangtze River Estuary (YRE) is one of the world’s largest river-tidal systems with rapidly changing hydrology and morphology following the construction of multiple dams. The effects of dam construction may extend to the region close to the coast, where channel stability depends on the asymmetry of the tide. Here, we focus on the possible effects of changing discharge regimes on tidal asymmetry in the YRE. Specifically, we focus on the difference in duration between ebb and flood, quantified as tidal duration asymmetry, because it has strong implications for residual sediment transport and can be derived from available water level data. To cope with nonstationary tides under the influence of a time-varying river discharge, a nonstationary harmonic analysis tool (NS_TIDE) is applied to explore the spatiotemporal variations in tidal duration asymmetry, under the influence of different combinations of tidal constituents. Tidal duration asymmetry initially increases, then slightly decreases, in an upstream direction. It experiences significant seasonal variations in response to rapidly varying discharge: tides are more asymmetric upstream of Zhenjiang in the dry season and more asymmetric downstream in the wet season. The combined effects of discharge regulation and morphological changes cause seasonal alterations in tidal duration asymmetry. In the wet season, reduced river discharge caused by water storage and climate change enhance the asymmetry upstream (+11.74% at Wuhu, +7.19 at Nanjing) while the asymmetry is weakened downstream (−2.90% at Zhenjiang, −7.19 at Jiangyin) following the TGD’s operation. Downstream channel erosion caused by post-TGD lower sediment loads has become the dominant factor weakening tidal asymmetry in most parts of the YRE in the dry season. Understanding these evolutions of tidal duration asymmetry under the hydrological and morphological effects has important implications for the management of estuarine ecosystem and navigation.

## Introduction

Dams are built for various purposes, such as irrigation, flood control and power generation^1^. The various impacts of dams have been subject to study and debated for decades. The changes in river and sediment discharges due to dam constructions have long been recognized as far-reaching^2–4^. Dams play an important role in eliminating peak river discharge and stabilizing low river discharge^5^. Beyond discharge regulation, dams also lead to a drop in sediment loads, thereby impacting estuarine morphology^4,6–9^, which is crucially dependent on tidal asymmetry^10,11^. Here, we focus on tidal duration asymmetry, which quantifies the periodic difference between the durations of falling and rising tidal water level^12,13^. Tidal duration asymmetry characterizes flood and ebb dominance^14–16^, and can directly be derived from long-term water level data.

Tidal asymmetry can be produced when tides propagate into shallow-water areas, where tidal distortion results in rising high-frequency harmonic constituents^14,17^. The physical mechanisms responsible for tidal duration asymmetry are represented by the nonlinear effects related to tidal interactions among constituents and irregular estuarine topography^13,14,18,19^. Tides can be strongly modulated by river flow and have a complex behavior in estuaries^20,21^. Rapidly varying river discharges can generate significant nonstationary features^22–24^, characterized by tidal attenuation and tidal energy transfer between tidal frequencies as a frictional effect^25–29^. Thus, tide-river interactions can significantly influence tidal duration asymmetry^23^. When tidal elevation and velocity are near quadrature, the tidal duration asymmetry can be related to flow velocity asymmetry^12,30,31^, which has an important influence on sediment transport^17,32–34^. Tidal duration asymmetry also plays an important role in navigation and ecosystems by affecting the fluctuations in water levels and bed friction^22^.

It is common practice to evaluate tidal asymmetry by the harmonics of astronomical tides using the amplitude ratio between the semidiurnal tide M_2_ and its first harmonics M_4_ (M_4_/M_2_) to quantify the degree of distortion^15^; the phase difference (2M_2_ − M_4_) is used as a metric to reflect the direction of tidal asymmetry^12–14,32,34,35^. This approach can be extended to evaluate the contributions of triad combinations of tidal constituents, such as K_1_/O_1_/M_2_, which is important in mixed tidal regimes^18,19^. Song *et al*.^36^ followed the approach taken by Nidzieko^31^ and generalized the skewness metric to determine the contributions of different tidal interactions, where the tidal combinations have a frequency relationship of $2{\omega }_{1}={\omega }_{2}$ for pairs or ${\omega }_{1}+{\omega }_{2}={\omega }_{3}$ for triads, such as K_1_/O_1_/M_2_.

China’s Yangtze River Estuary (YRE) is a natural system whose hydrodynamics exhibit strong spatiotemporal variations, making it an excellent location to investigate estuarine tidal dynamics. Although the characteristics of tidal propagation in the YRE have been widely studied^37,38^, the effects of river discharge have long been neglected and remain a challenge to understand nonstationary tidal dynamics. Recently, increasing attention has been paid to the responses of tidal dynamics to varying river discharge in the YRE^23,39–44^, but few of those have described the long-term evolutions of tidal properties. In particular, it is of interest to establish the contrast before and after the operation of the Three Gorges Dam (TGD), which is the world’s largest river damming project. Its impacts have raised public concern following rapid economic development and increasing awareness of possible negative impacts for natural resources and the environment^45–47^.

Previous studies have focused on water discharge^48,49^, sediment load^50–52^, estuarine Suspended Sediment Concentration^53^, delta evolution^4,45,54,55^ and sediment grain size^56–58^. To mitigate floods and droughts in the middle and lower regions of the Yangtze River, the TGD reduces the peak discharge in the wet season whereas increases discharge during the dry season^48,49,59^. The riverine sediments are mostly trapped in reservoirs, causing severe channel erosion in the subaqueous delta^4,60,61^. These seasonal discharge regulation and morphological changes significantly affect the tidal dynamics in the YRE. Little is known about the impact of the dam on estuarine tidal dynamics. Zhang *et al*.^62^ used a numerical model to study the interactions between tidal waves and river discharge with reference to the TGD’s impact focusing on different tidal constituents. The dominant M_2_ tide has decreased from the dry season to wet season. Cai *et al*.^63^ investigated the effects of the TGD on spatiotemporal variations in tide-river dynamics, with the largest change occurring in autumn. In addition, the YRE has undergone significant morphological changes under the influences of human activities such as dam constructions and land use patterns^45,56,61,64,65^, which also affect the tidal duration asymmetry in the system. Hence, a better understanding of the relationship between tidal duration asymmetry and river discharge and morphology with respect to the TGD is still missing.

In this study, the changes in tidal duration asymmetry due to different tidal combinations are investigated in the YRE with respect to the large and strongly variable river discharge and morphological changes related to the effects of the TGD. A nonstationary harmonic model (NS_TIDE) is applied to long-term observational data in the YRE to assess the spatiotemporal variations in the tidal duration asymmetry in the pre- and post-TGD periods. The seasonal evolution of tidal duration asymmetry may inform other tidal estuaries under the impact of large dams, thus providing a basis for further understanding these dynamics elsewhere in the world.

## Methods

### Study area

The Yangtze River, with a drainage area of 1.8 × 10^6^ km^2^, is the largest river in southern Asia^4^. The YRE is located on the eastern China coast. As the major channel connecting the Yangtze River and East China Sea, the YRE is influenced by moderate tides and strongly varying river discharges^66^. The YRE has a semidiurnal tidal regime ($({a}_{{K}_{1}}+{a}_{{O}_{1}})/({a}_{{M}_{2}}+{a}_{{S}_{2}}) < 0.25$, in which *a* represents tidal amplitude), where M_2_ is the dominant tide and followed by S_2_, K_1_ and O_1_. Under the intermediate river flow and tidal conditions, Jiangyin is the landward limits of tidal current reversal (240 km away from the mouth), while the tidal wave propagates upstream until Datong (645 km away from the mouth).

**Figure 1:**
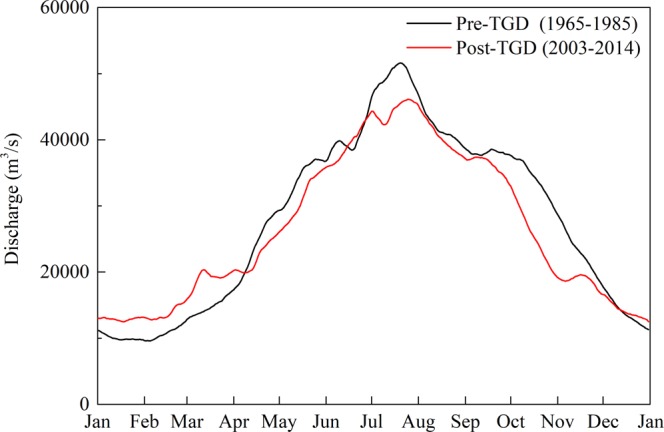
Yangtze River discharge at the Datong gauging station before and after the operation of the TGD.

The YRE has an annual mean river discharge of approximately 27,500 m^3^/s at Datong for the whole study period (1965–1985 & 2003–2014). However, seasonal discharge varies substantially under the influence of the summer Asian monsoon, with low discharge during the dry season (November to April) and high discharge during the wet season (May and October) (Fig. 1). In addition, river discharge at Datong experiences a significant seasonal regulation after the operation of the TGD, characterized by a decrease of 3430 m^3^/s (altered from 39611 m^3^/s to 36181 m^3^/s) during the wet season and a slight increase of 231 m^3^/s (altered from 16210 m^3^/s to 16441 m^3^/s) during the dry season (Fig. 1). Those changes can be attributed to the combined effects of the TGD regulation and climate changes that have occurred in the Yangtze basin^67,68^.

### Data overview

**Figure 2:**
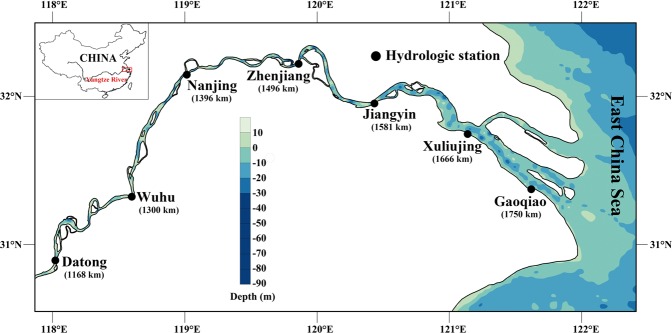
Map of the Yangtze River Estuary and the locations of hydrological stations. The numbers in brackets are distance downward the Three Gorges Dam. Note: the maps were created using Surfer 13 (www.goldensoftware.com/products/surfer).

The YRE, as a subject of our analysis, has been intensively monitored for water levels since the mid-1950s. Available hydrological data were divided into 2 periods according to the TGD operation in 2003: the pre-TGD period (1965–1985, before the impoundment of the TGD) and the post-TGD period (2003–2014, after the operation of TGD). The period 1986–2002 was not included in our analysis due to lack of data. Water level data spanning the two periods at six hydrological stations which are located throughout the estuary were collected. The location of these stations in the YRE are presented in Fig. 2 and the data periods of all stations are listed in Table 1. Water level data of six stations were adjusted to Wusong mean sea level for the analysis. For the purpose of harmonic analysis, the original high-low water level data are interpolated into one-hour intervals (Appendix A). In addition, a series of daily-averaged river discharge data for the pre and post periods at Datong are used to establish the impact of river discharge. All the data are collected from the Hydrological Yearbooks of the People’s Republic of China, published by the Yangtze Hydrology Bureau of China.

**Table 1:** Location of hydrological stations and the corresponding periods of valid data.

No.	Station name	Location		Data period	Distance to TGD (km)
		Longitude (E)	Latitude (N)		
1	Datong	117°38′	30°46′	1965–1985, 2003–2014	1115
2	Wuhu	118°21′	31°21′	1965–1985, 2003–2014	1239
3	Nanjing	118°43′	32°05′	1965–1985, 2003–2014	1335
4	Zhenjiang	119°26′	32°13′	1965–1985, 2003–2014	1435
5	Jiangyin	120°18′	31°57′	1965–1985, 2003–2014	1520
6	Xuliujing	120°57′	31°46′	1982–1985, 2003–2014	1605
7	Gaoqiao	121°33′	31°23′	1965–1985, 2003–2014	1685

### Methodology

#### Nonstationary harmonic analysis method

Harmonic analysis (HA), which determines tidal amplitudes and phases by a least squares regression analysis, traditionally assumes that tides are perfectly stationary^69,70^. However, tides in estuaries are affected not only by astronomical tidal forcing but also nonlinear interactions generating subharmonics (e.g. river flow, channel geometry and friction)^71^. The traditional HA is not effective enough for the analysis of river tides when the river flow is strongly variable. A nonstationary harmonic analysis method (NS_TIDE) is developed based on T_TIDE^72^ by embedding the nonstationary forcing into the tidal basis functions^73^. It has been applied to the Columbia River Estuary^73^ and St. Lawrence River^74^ to prove that it can be a useful method in analyzing the nonstationary water level record reflected by external forcing (oceanic tide and river flow) and nonlinear interactions. NS_TIDE can effectively distinguish frequencies within tidal bands without sacrificing resolution in the time domain or data at the end of time series.

The NS_TIDE method developed by Matte *et al*.^73,74^ is applied to estimate spatial and temporal evolutions of tidal harmonic constants. In this nonstationary tidal harmonic model, the total tidal height is given as:

1 $$\begin{array}{c}h(t)={c}_{0}+{c}_{1}{Q}^{{p}_{s}}(t-{t}_{Q})+{c}_{2}\frac{{R}^{{q}_{s}}(t-{t}_{R})}{{Q}^{{r}_{s}}(t-{t}_{Q})}\\ +\mathop{\sum }\limits_{k=1}^{n}\left[\begin{array}{c}\left({d}_{0,k}^{(c)}+{d}_{1,k}^{(c)}{Q}^{{p}_{f}}(t-{t}_{Q})+{d}_{2,k}^{(c)}\frac{{R}^{{q}_{f}}(t-{t}_{R})}{{Q}^{{r}_{f}}(t-{t}_{Q})}\right)\cos (t)\\ +\left({d}_{0,k}^{(s)}+{d}_{1,k}^{(s)}{Q}^{{p}_{f}}(t-{t}_{Q})+{d}_{2,k}^{(c)}\frac{{R}^{{q}_{f}}(t-{t}_{R})}{{Q}^{{r}_{f}}(t-{t}_{Q})}\right)\sin (t)\end{array}\right]\end{array}$$

where ${c}_{0}$, ${c}_{1}$ and ${c}_{2}$ denote the coefficients of stage model; *Q* represents the upstream river flow; *R* represents the greater diurnal tidal range at the reference station; ${p}_{s}$, ${q}_{s}$, ${r}_{s}$, ${p}_{f}$, ${q}_{f}$ and ${r}_{f}$ are the exponents for each station and frequency band, which are obtained by an iterative procedure, where the subscripts s and f refer to the stage and tidal-fluvial models, respectively; ${d}_{0,k}$, ${d}_{1,k}$ and ${d}_{2,k}$ are the model coefficients for the tidal-fluvial model, while the superscripts $c$ and $s$ denote the cosine and sine terms; ${t}_{Q}$ and ${t}_{R}$ are the time-lags capturing the average propagation time of waves to the station, determined by calculating the maximum correlation between *Q* or *R* time series and the observed data.

#### Model performance

**Figure 3:**
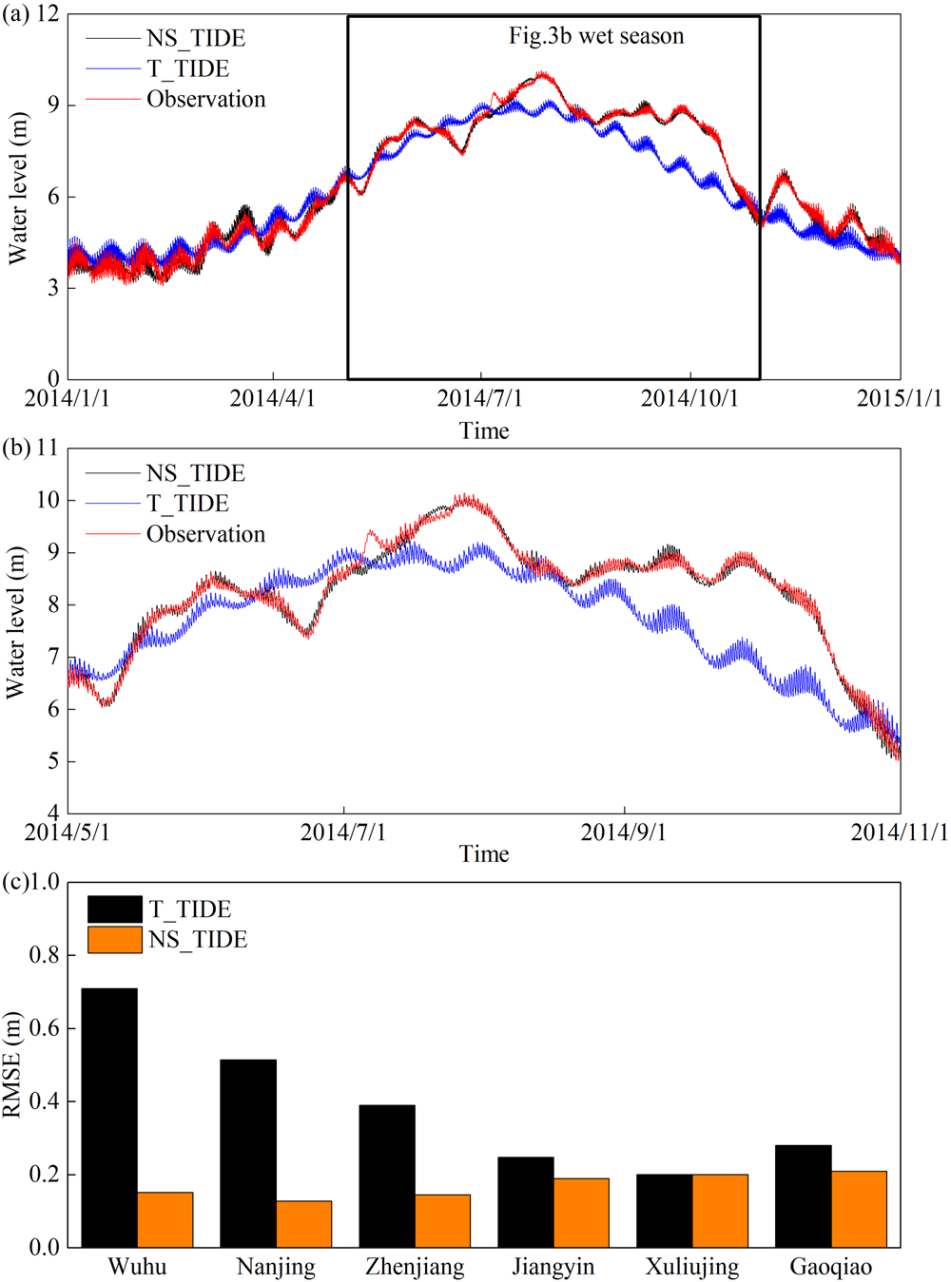
(**a**) Comparison of observed water level at Wuhu in 2014 with hindcasts obtained from the NS_TIDE and T_TIDE models; (**b**) zoom-in of data in wet season; (**c**) Root-mean-square error (RMSE) of water level hindcasts at 6 hydrological stations ordered from upstream to downstream.

The ‘harmonic constants’ are not constant over time in the YRE, where the river flow is strongly variable and the tidal signal is highly nonstationary. Hence, the observations at Wuhu in 2014 with hindcasts obtained by the NS_TIDE and T_TIDE models are compared to evaluate the effectiveness and applicability of NS_TIDE in the YRE (Fig. 3a). T_TIDE is unable to resolve seasonal fluctuations related to variable river discharge, while NS_TIDE is far more accurate, especially during the wet season (Fig. 3b). The root-mean-square errors (RMSE) values are calculated at six gauging stations (Fig. 3c). The RMSE for both models are comparable at downstream stations, while the RMSE for T_TIDE increase sharply upstream under the significant influence of river discharge. For T_TIDE, the hindcast explains 86.44% of the original signal variance and has a RMSE of 0.710 m at Wuhu. Results from NS_TIDE sharply improves compared to T_TIDE: the hindcast explains 99.64% of the original signal variance and has an RMSE of 0.146 m. Thus, NS_TIDE is applied to further analyses in this study.

#### Tidal skewness metric

The skewness metric is used in the present study to analyze the characteristics of overall tidal duration asymmetry and to determine the contributions of different combinations of tidal constituents. This metric depends on the tidal amplitudes (a) and phases (*φ*), as well as frequencies of tidal constituent (*ω*). As indicated in Song *et al*.^36^, the skewness is obtained as:

2 $${\gamma }_{2}=\frac{\frac{3}{4}{a}_{1}^{2}{\omega }_{1}^{2}{a}_{2}{\omega }_{2}\,\sin (2{\varphi }_{1}-{\varphi }_{2})}{{\left[\frac{1}{2}({a}_{1}^{2}{\omega }_{1}^{2}+{a}_{2}^{2}{\omega }_{2}^{2})\right]}^{3/2}}$$

and

3 $${\gamma }_{3}=\frac{\frac{3}{2}{a}_{1}{\omega }_{1}{a}_{2}{\omega }_{2}{a}_{3}{\omega }_{3}\,\sin ({\varphi }_{1}+{\varphi }_{2}-{\varphi }_{3})}{{\left[\frac{1}{2}({a}_{1}^{2}{\omega }_{1}^{2}+{a}_{2}^{2}{\omega }_{2}^{2}+{a}_{3}^{2}{\omega }_{3}^{2})\right]}^{3/2}}$$

where $2{\omega }_{1}={\omega }_{2}$ (pairs) in Eq. (2) and ${\omega }_{1}+{\omega }_{2}={\omega }_{3}$ (triads) in Eq. (3), respectively. The contribution of different combinations of two or three tidal constituents to total tidal skewness is calculated as follows:

4 $${\beta }_{2}=\frac{\frac{3}{4}{a}_{1}^{2}{\omega }_{1}^{2}{a}_{2}{\omega }_{2}\,\sin (2{\varphi }_{1}-{\varphi }_{2})}{{\left(\frac{1}{2},\mathop{\sum }\limits_{i=1}^{N},{a}_{i}^{2}{\omega }_{i}^{2}\right)}^{3/2}}={\gamma }_{2}\cdot {\left(\frac{{a}_{1}^{2}{\omega }_{1}^{2}+{a}_{2}^{2}{\omega }_{2}^{2}}{\mathop{\sum }\limits_{i=1}^{N}{a}_{i}^{2}{\omega }_{i}^{2}}\right)}^{3/2}$$

for pairs or

5 $${\beta }_{3}=\frac{\frac{3}{2}{a}_{1}{\omega }_{1}{a}_{2}{\omega }_{2}{a}_{3}{\omega }_{3}\,\sin ({\varphi }_{1}+{\varphi }_{2}-{\varphi }_{3})}{{\left(\frac{1}{2}\mathop{\sum }\limits_{i=1}^{N}{a}_{i}^{2}{\omega }_{i}^{2}\right)}^{3/2}}={\gamma }_{3}\cdot {\left(\frac{{a}_{1}^{2}{\omega }_{1}^{2}+{a}_{2}^{2}{\omega }_{2}^{2}+{a}_{3}^{2}{\omega }_{3}^{2}}{\mathop{\sum }\limits_{i=1}^{N}{a}_{i}^{2}{\omega }_{i}^{2}}\right)}^{3/2}$$

for triplets. Thus, the total tidal skewness is calculated as the summation of the individual *β*.

6 $${\gamma }_{N}={\sum \beta }_{2}+{\sum \beta }_{3}$$

The sign of tidal skewness reflects the direction of tidal duration asymmetry and the value can reflect the degree of distortion. For ${\gamma }_{N} < 0$, the falling tide duration is shorter than the rising tide duration. Conversely, the positive value for tidal skewness (${\gamma }_{N} > 0$) indicates shorter rising tide duration.

#### Relative sensitivity coefficient (RSC)

The tidal skewness evaluates the contributions of each combination to tidal duration asymmetry based on the amplitudes, phases and frequencies of the constituents. The non-dimensional relative sensitivity coefficient (RSC)^75^ is introduced to study the sensitivity of tidal skewness contributed by different tidal combinations to the attenuation of the corresponding tidal amplitude. The expression of the RSC reads:

7 $${S}_{a}=\mathop{\mathrm{lim}}\limits_{\varDelta A\to 0}(\frac{\Delta \gamma /\gamma }{\Delta a/a})=\frac{\partial \gamma }{\partial a}\cdot \frac{a}{\gamma }$$

Generally, the RSCs for combination of two or three constituents are obtained as follows:

8 $${S}_{{a}_{1}}=\frac{\partial \gamma }{\partial {a}_{1}}\cdot \frac{{a}_{1}}{\gamma }=\frac{2{a}_{2}^{2}{\omega }_{2}^{2}-{a}_{1}^{2}{\omega }_{1}^{2}}{{a}_{1}^{2}{\omega }_{1}^{2}+{a}_{2}^{2}{\omega }_{2}^{2}}$$

9 $${S}_{{a}_{2}}=\frac{\partial \gamma }{\partial {a}_{2}}\cdot \frac{{a}_{2}}{\gamma }=\frac{{a}_{1}^{2}{\omega }_{1}^{2}-2{a}_{2}^{2}{\omega }_{2}^{2}}{{a}_{1}^{2}{\omega }_{1}^{2}+{a}_{2}^{2}{\omega }_{2}^{2}}$$

for the combination of two constituents, and

10 $${S}_{{a}_{1}}=\frac{\partial \gamma }{\partial {a}_{1}}\cdot \frac{{a}_{1}}{\gamma }=\frac{{a}_{2}^{2}{\omega }_{2}^{2}+{a}_{3}^{2}{\omega }_{3}^{2}-2{a}_{1}^{2}{\omega }_{1}^{2}}{{a}_{1}^{2}{\omega }_{1}^{2}+{a}_{2}^{2}{\omega }_{2}^{2}+{a}_{3}^{2}{\omega }_{3}^{2}}$$

11 $${S}_{{a}_{2}}=\frac{\partial \gamma }{\partial {a}_{2}}\cdot \frac{{a}_{2}}{\gamma }=\frac{{a}_{1}^{2}{\omega }_{1}^{2}+{a}_{3}^{2}{\omega }_{3}^{2}-2{a}_{2}^{2}{\omega }_{2}^{2}}{{a}_{1}^{2}{\omega }_{1}^{2}+{a}_{2}^{2}{\omega }_{2}^{2}+{a}_{3}^{2}{\omega }_{3}^{2}}$$

12 $${S}_{{a}_{3}}=\frac{\partial \gamma }{\partial {a}_{3}}\cdot \frac{{a}_{3}}{\gamma }=\frac{{a}_{1}^{2}{\omega }_{1}^{2}+{a}_{2}^{2}{\omega }_{2}^{2}-2{a}_{3}^{2}{\omega }_{3}^{2}}{{a}_{1}^{2}{\omega }_{1}^{2}+{a}_{2}^{2}{\omega }_{2}^{2}+{a}_{3}^{2}{\omega }_{3}^{2}}$$

for the combination of three constituents, where ${a}_{1}$, ${a}_{2}$ and ${a}_{3}$ refer to tidal amplitude, ${\omega }_{1}$, ${\omega }_{2}$ and ${\omega }_{3}$ are the tidal frequencies. The positive/negative values of RSCs typically suggest the positive/negative correlation between tidal skewness and amplitudes. Specifically, an ${S}_{a}$ value of 0.5 indicates that a 10% decrease of the tidal amplitude *a* can make the corresponding tidal skewness ($\gamma$) decrease by 5%. A larger absolute value of the coefficient (RSC) is interpreted as a more significant effect of the variable A on the corresponding tidal duration asymmetry.

## Results

### Spatial patterns of tidal dynamics

#### Spatial patterns of tidal amplitudes and phases

The spatial variations in the amplitudes and phases of the seven main tidal constituents (K_1_, O_1_, M_2_, S_2_, M_4_, MS_4_ and M_6_) at the six hydrological stations modelled by NS_TIDE are presented in Table 2. The diurnal (K_1_ and O_1_) and semi-diurnal (M_2_ and S_2_) constituents show a landward decrease and the amplitudes are maximum at the most seaward station (Gaoqiao). The amplitudes of K_1_ and O_1_ are in the range of 0.04–0.24 m and 0.04–0.16 m, respectively. As the dominant constituent in the YRE, M_2_ is larger than other constituents with an amplitude ranging from 0.11–1.12 m. The S_2_ amplitude is 0.04–0.29 m, about 1/4-1/3 of the M_2_ amplitude. According to Godin^28^, the decay of tidal amplitudes is frequency-dependent: the tidal constituents with lower frequencies decay more slowly than those with higher frequencies. The development of tidal phases also seems to agree with this trend.

**Table 2:** Tidal amplitudes (a) and phases (p) of main constituents in the YRE.

	K_1_		O_1_		M_2_		S_2_		M_4_		MS_4_		M_6_		K
	a(m)	p(°)	a(m)	p(°)	a(m)	p(°)	a(m)	p(°)	a(m)	p(°)	a(m)	p(°)	a(m)	p(°)	
Wuhu	0.043	75	0.037	40	0.111	47	0.041	103	0.020	360	0.018	41	0.005	300	0.522
Nanjing	0.079	29	0.063	330	0.243	324	0.084	26	0.042	200	0.038	245	0.011	78	0.432
Zhenjiang	0.119	354	0.092	293	0.433	261	0.130	320	0.084	73	0.075	122	0.022	245	0.376
Jiangyin	0.173	295	0.121	237	0.769	160	0.236	220	0.137	234	0.127	281	0.035	307	0.293
Xuliujing	0.207	253	0.142	199	0.924	86	0.250	145	0.149	91	0.136	133	0.028	81	0.298
Gaoqiao	0.244	215	0.162	157	1.118	16	0.285	14	0.123	308	0.106	349	0.020	241	0.289

When tides propagate into the YRE, they are significantly distorted, as expressed in the generation of shallow-water constituents, such as M_4_, MS_4_ and M_6_. This distortion is characterized by an initial increase and subsequent rapid decrease of shallow water tidal amplitudes further upstream. M_4_ varies similarly to MS_4_, likely due to their close frequencies. The amplitudes of M_4_ and MS_4_ increase toward the lower reaches of the estuary, peaking at 0.15 m and 0.14 m, respectively. These two quarter-diurnal constituents are suppressed at Xuliujing, where frictional damping gradually becomes dominant over shallow-water amplification. Similar patterns are noted for M_6_, with a peak amplitude of 0.04 m at Jiangyin.

The character of the tides can be quantified by the tidal form number *F*, expressed as $F=\frac{{K}_{1}+{O}_{1}}{{M}_{2}+{S}_{2}}$^76,77^. It varies in the range of 0.289–0.522 for the YRE, indicating that the YRE can be classified as a mixed semidiurnal tidal regime (0.25 < *F* < 1.5). The tidal form number generally show an increasing trend when the tidal wave propagates upstream, possibly because the semidiurnal constituents fade out faster than the diurnal constituents (which have higher frequencies).

#### Spatial pattern of the overall tidal duration asymmetry

**Figure 4:**
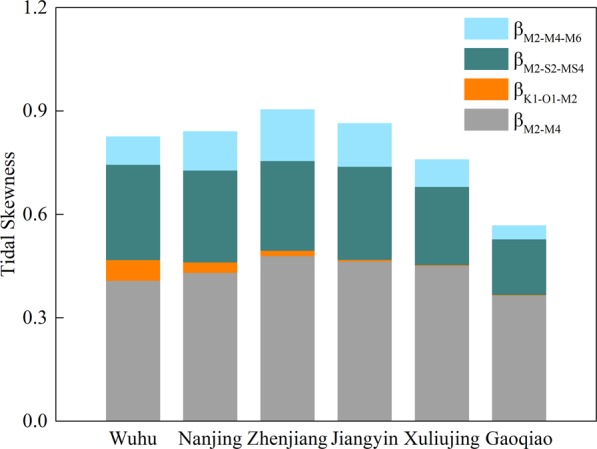
Total tidal skewness (*β*) contribution from the four major constituent combinations for each of the six hydrological stations.

The tidal skewness (*β*) offers a concise and quantitative way to describe the overall tidal duration asymmetry. Four major tidal combinations are selected for this purpose (Fig. 4). Tidal duration asymmetry originating from the interactions of both astronomical tides and shallow-water constituents in the YRE is predominantly flood dominant, as reflected by the positive values of *β* at the six stations. The overall flood asymmetry has a significant landward increase and is followed by a slight decrease upstream.

Generally, nonlinear tidal interactions are the main drivers of tidal duration asymmetry within the YRE, of which M_2_/M_4_ is the most important contributor with the largest magnitude (Fig. 4), followed by M_2_/S_2_/MS_4_, which accounts for ~30% of the total tidal skewness. M_2_/M_4_/M_6_ ranks third. Overall, the asymmetry induced by these nonlinear effects first increases and then decreases, in line with the spatial evolution of the overall tidal duration asymmetry. In comparison, the K_1_/O_1_/M_2_ triad has a relatively small skewness value and retain a slight increase from the lower reaches to the tidal river. This indicates that the tide entering the channel is flood-dominant within the YRE. However, K_1_/O_1_/M_2_ in the YRE behaves differently from other tidal systems, such as the southern California coast^31,78^ and the Pearl River Delta^16^, where tidal duration asymmetry is ebb-dominant prior to nonlinear tidal distortions within the tidal systems. This difference may be related to channel geometrical properties and the interactions between tidal constituents^31^.

### Seasonal variations in tidal dynamics

#### Seasonal variations in tidal amplitudes and phases

**Figure 5:**
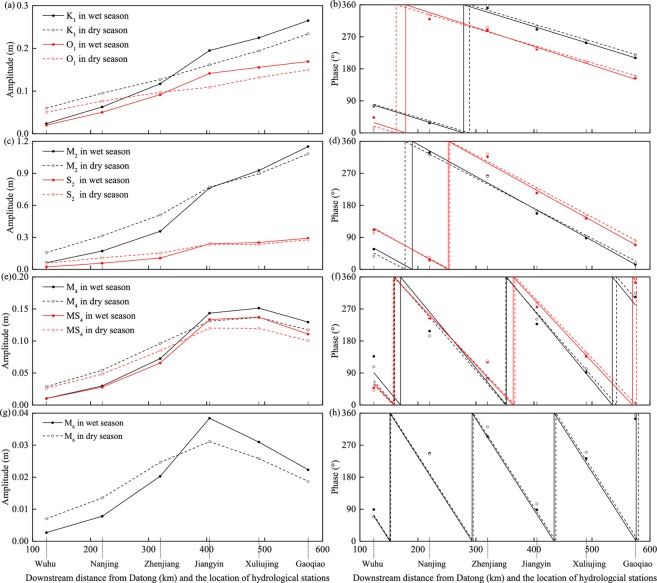
Seasonal dynamics of tidal amplitude and phase for (**a–d**) the main astronomical (K_1_, O_1_, M_2_ and S_2_) and (**e–h**) shallow-water constituents (M_4_, MS_4_ and M_6_) at six hydrological stations.

The variations in tidal amplitudes and phases at the six gauging stations are shown in Fig. 5 for the wet season and the dry season. The astronomical tidal constituents show significant seasonal variations. Smaller amplitudes are found upstream in the wet season, likely due to the higher river discharge that attenuate astronomical tides via friction. In addition, the seasonal differences in astronomical tidal amplitudes are more significant upstream than in the middle part of the YRE, showing a pattern opposite to that observed in the lower reaches of the YRE, where increasing discharge raises the water level and increased the tidal amplitude^74^. The trends in the tidal phase reflect the speed of tidal propagation along the river. Increasing river discharge delays the tidal propagation, leading to an increase in the astronomical tidal phase in the wet season at the upstream stations. In the lower reaches of the YRE, smaller astronomical tidal phases occur in the wet season due to deeper water as the river discharge increases^74^.

As for the shallow-water constituents, the high river discharge during the wet season increases the tidal amplitudes in the downstream region by enhancing the nonlinear effect and stimulating tidal energy transfer from astronomical tides to shallow-water tides (Fig. 5). As tides propagate to the upper reach of the YRE, smaller tidal amplitudes occur in the wet season, when higher river discharge leads to a more significant tidal damping process by enhancing the frictional effects. The variations in shallow-water constituent phases show similar characteristics as the astronomical constituents on a seasonal scale: they increase because of larger discharge in the wet season, especially at the upstream stations.

#### Seasonal variations in tidal duration asymmetry

**Figure 6:**
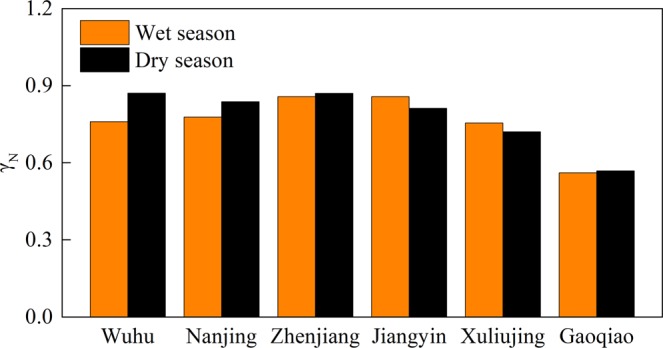
Comparison of tidal skewness during the flood and dry season for the six hydrological stations.

Tidal skewness displays significant seasonality variations (Fig. 6). In the upper reaches, tidal duration asymmetry in the dry season is more significant than in the wet season. In addition, a significant difference (${\gamma }_{{\rm{dry}}{\rm{season}}}-{\gamma }_{{\rm{wet}}{\rm{season}}}=0.111$, corresponding to 14.67%) occurs at Wuhu, while a slight difference (${\gamma }_{{\rm{dry}}{\rm{season}}}-{\gamma }_{{\rm{wet}}{\rm{season}}}=0.013$, corresponding to 1.46%) occurs at Zhenjiang, which suggests that the effects of river discharge are more significant upstream. However, the opposite pattern occurs at the downstream stations, except at Gaoqiao. This may reflect the enhanced effects of nonlinearities due to increasing discharge during the wet season^41,79,80^, leading to stronger shallow-water tides and weaker astronomical tides. The generation of forced constituents and the decay of basic constituents are the major factors influencing the generation of tidal duration asymmetry in the YRE, such that flood asymmetry is enhanced at the downstream stations in the wet season. Around the estuary mouth, the effects of discharge are less significant, making the seasonal variations least obvious at Gaoqiao.

### Changes in the tidal duration asymmetry between the pre- and post-TGD periods

**Figure 7:**
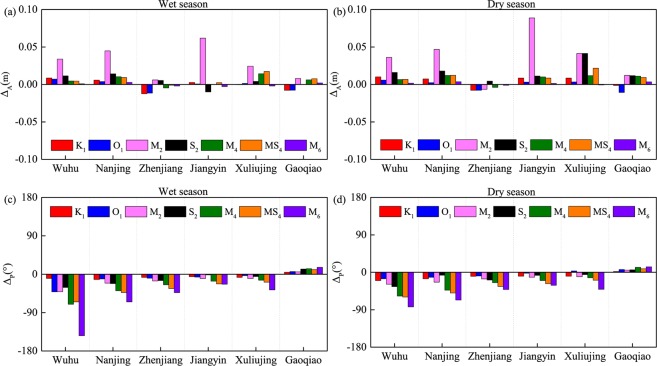
Differences in tidal amplitudes and phases for all six hydrological stations in the wet (**a,c**) and dry (**b,d**) season before and after TGD’s operation.

To estimate the impacts of the TGD on the tidal dynamics in the YRE, the differences between the pre-YGD and post-TGD periods are evaluated in Fig. 7 (${\Delta }_{a}={a}_{post-TGD}-{a}_{pre-TGD}$ and ${\Delta }_{p}={p}_{post-TGD}-{p}_{pre-TGD}$, where *a* and *p* refer to the tidal amplitude and phase, respectively). During the wet season, the values of ${\Delta }_{a}$ for the astronomical tides (K_1_, O_1_, M_2_ and S_2_) at most stations are positive because of the significant decrease in the river discharge caused by water storage (Fig. 7a). Similarly, shallow-water tidal amplitudes increase at the upstream stations after the TGD’s operation (Fig. 7b). The strength of amplitude increase varies significantly along the channel, with the smallest ${\Delta }_{a}$ occurring at the most seaside station (Gaoqiao) because the impacts of river discharge are smallest at the mouth of the estuary. However, the largest differences do not necessarily occur in the upper reaches due to the significant attenuation over longer distances. Consequently, Jiangyin (in the middle reach) displays the most significant amplitude variations under the effects of the regulated river discharge. This is consistent with Zhang *et al*.^62^, in which the numerical modelling shows that the largest differences occur in the middle part of the YRE. The speed of tidal propagation can be seen in variations of tidal phases. As for the changes in tidal phases during the wet season, reduced river discharge allows tides to propagate further into the YRE, characterized by smaller tidal phases in the post-TGD period, especially for the tidal constituents with higher frequencies. With increased river discharge, the largest decrease in tidal phases occurs at Wuhu.

The YRE experienced not only discharge regulation but also the morphological changes. The impact of morphological changes on tidal dynamics gradually becomes dominant in the dry season, when discharge regulation is insignificant. It can be seen from Fig. 1 that river discharge increases by only 231 m^3^/s (corresponding to 1.4%) after the operation of the TGD. Hence, an understanding of the morphological impacts on tidal dynamics becomes increasingly important. The TGD’s operation lead to a dramatic reduction in sediment supply in the YRE^56^, increasing erosion^61^ and causing channel deepening, which reduces bottom friction^79,81,82^. It reduces tidal attenuation along the channel^41^, resulting in increased tidal amplitudes at most stations after the TGD’s operation (Fig. 7c). The deepening channel also makes it easier for the tides to enter the estuary, as reflected by the decreased tidal phase (Fig. 7d). A weakened friction caused by channel deepening causes the tides to propagate faster into the estuary after the operation of the TGD. Because of the intense attenuation effects of high river discharge, the tidal amplitudes in the wet season are smaller than in the dry season, so that the amplitude change is insignificant. As a result, the amplitude differences are relatively small in the wet season. Changes in the tidal phase during the wet season are more significant under the combined effects of discharge regulation and morphological changes.

**Figure 8:**
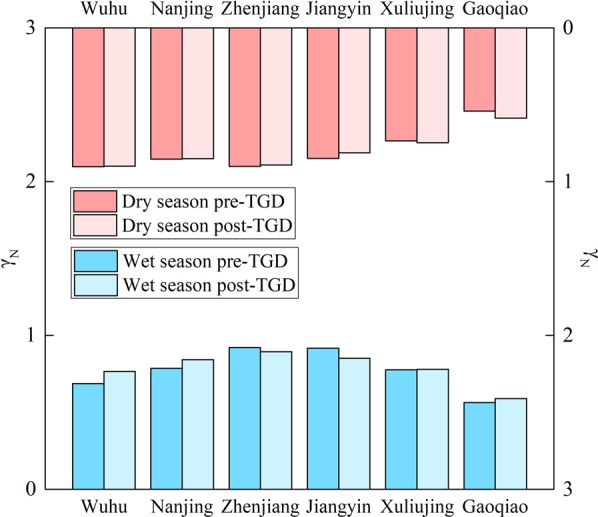
Along-river variations in mean tidal skewness ${\gamma }_{N}$ during the pre- and post-TGD periods.

During the wet season, the post-TGD tidal skewness increases (compared to pre-TGD values) in the upper reaches at Wuhu (0.081) and Nanjing (0.057), but decreases in the middle reaches. These obvious seasonal differences are largely due to reduced river discharge caused by water storage. The difference between the two periods is negligible at Xuliujing, indicating that the impact of the discharge regulation weakens with the distance from the TGD. Tidal skewness also varies significantly in the dry season (see red columns in Fig. 8). The tidal duration asymmetry is slightly weakened in the upper and middle reaches after the TGD’s operation in response to increasing channel depth, similar to the Pearl River Delta^16^ and the Ems Estuary^83^. The frictional effects are reduced with increasing water depth, which weakens tidal attenuation along the channel. Since the attenuation of principal tides is less significant than that of higher harmonics within the estuary, the flood asymmetry is thus reduced after the TGD’s operation. In contrast, the tidal duration asymmetry is enhanced at the downstream stations, where more complex morphological evolution occurs.

## Discussion

### Sensitivity of tidal amplitudes and phases to river discharge

**Figure 9:**
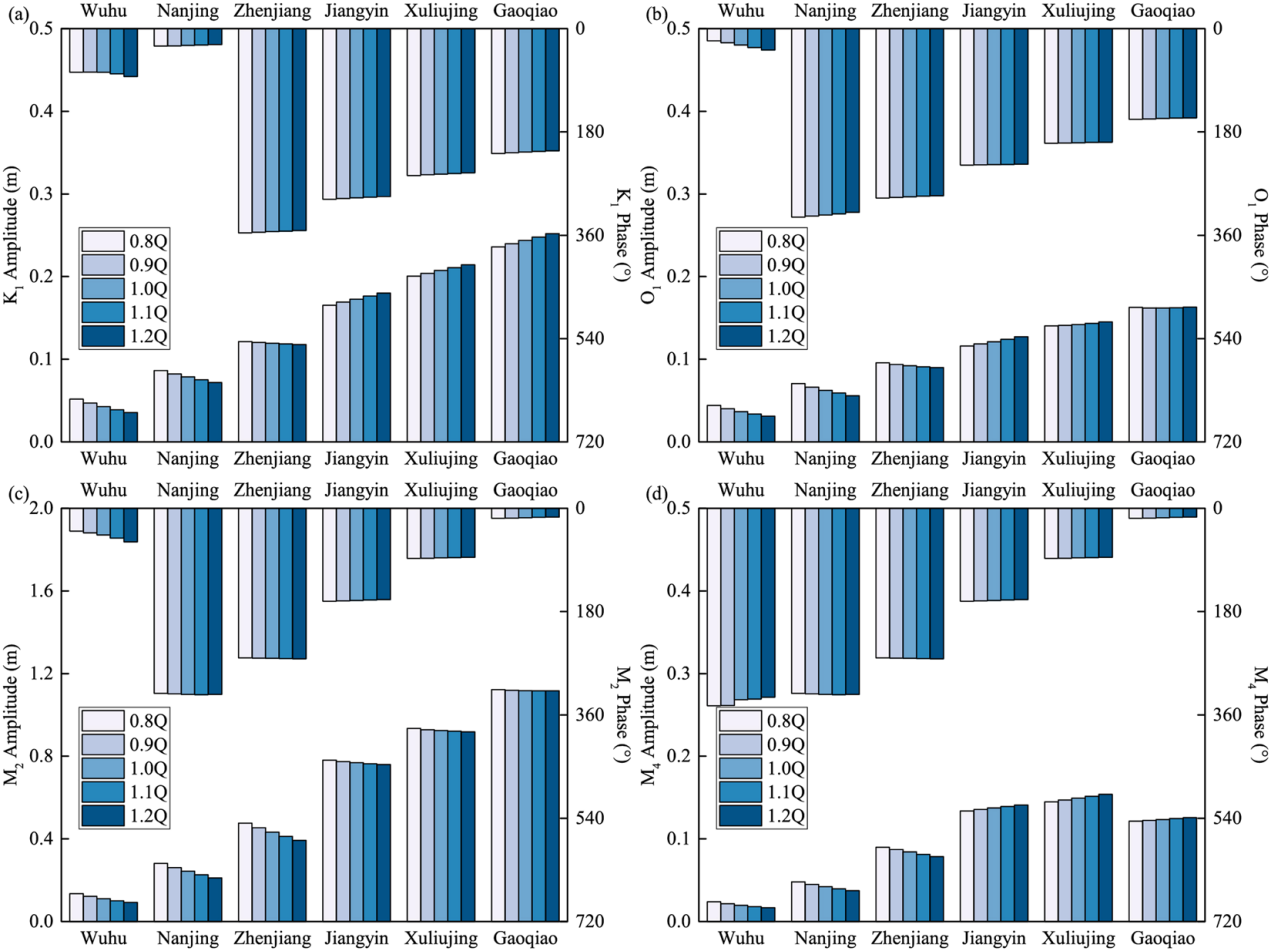
Sensitivity of tidal amplitudes (bottom column) and phases (top column) to different quantiles of river discharge for (**a**) K_1_, (**b**) O_1_, (**c**) M_2_ and (**d**) M_4_.

River discharge significantly influences tidal dynamics in the YRE, especially in the upper reaches^21,25,28,84^, and can be described as a frequency-dependent modulation of the tidal amplitudes and phases^28,85^. NS_TIDE is used to conduct a sensitivity analysis for tidal amplitude and phase to different quantiles (0.8, 0.9, 1.0, 1.1 and 1.2) of the river discharge, and to evaluate the impact of river discharges on tidal motion in the YRE (Fig. 9).

Tidal amplitudes and phases respond to varying river discharge with a distinct spatial pattern. The two dominant diurnal tides (K_1_ and O_1_) are damped by increasing discharges in most parts of the YRE (Fig. 9a,b). Although relatively larger K_1_ and O_1_ tidal phases are found in the upper parts of the estuary at higher discharges, their tidal phases remain stable. The increasing K_1_ amplitude downstream of Jiangyin is probably a result of increasing water depth from higher discharge, in accordance with the results of Matte *et al*.^74^ in the lower reaches of the St. Lawrence fluvial estuary. Tidal amplitudes become more sharply damped by the river discharge upstream of Zhenjiang, indicating a separation of the estuary into tide- and river-dominated sections between Zhenjiang and Jiangyin, possibly due to breaks in the bed morphology^81^ or rapid variation in bottom slope^74^. In contrast, O_1_ seems less dependent on morphology, possibly due to different effects of M_2_ on O_1_ relative to K_1_ under strong bottom frictions^86^.

The dominant semidiurnal constituent (M_2_) shows similar spatial variations as the diurnal constituents (K_1_ and O_1_) with respect to phases in most of the estuary (Fig. 9c). Slightly higher phases for M_2_ occur at higher discharge, implying that tidal propagation is delayed under the increasing influence of river discharge. The amplitudes of M_2_ decrease with increasing river discharge throughout the estuary, different from the two main diurnal constituents (K_1_ and O_1_) in the downstream region. At the upstream station (Wuhu), the M_2_ amplitudes for the 0.8 quantiles of discharge (0.8Q) decrease by 31.3% at the 1.2 quantiles (1.2Q), whereas at the most seaward station (Gaoqiao) the decrease is only 0.5%, suggesting that varying river discharge barely modulates tidal amplitudes at the mouth of the estuary. This implies that the correlation in the upper reaches is more sensitive than that in the lower reaches.

The sensitivity of the quarter-diurnal tide (M_4_) to river discharge to different quantiles of river discharge shows two clearly contrasting zones with marked changes in the M_4_ amplitudes around Jiangyin and Zhenjiang (Fig. 9d). When the tide enters the estuary, the M_4_ constituent is induced by friction, and its amplitude seems to be less sensitive to the varying discharge at Gaoqiao. As the tide propagates up the estuary, the increasing influence of discharge results in increasing M_4_ amplitudes and lower M_4_ phases at Xuliujing and Jiangyin, indicating an energy transfer from M_2_ to M_4_. Upstream of Jiangyin, M_4_ is attenuated at higher discharges with smaller amplitudes and larger phases along with the main tidal constituents. This spatially nonlinear behavior in response to varying discharge reveals the transition between tide- and river-dominated sections of the YRE.

### Impacts of tidal attenuation and phase shifting on tidal duration asymmetry

**Figure 10:**
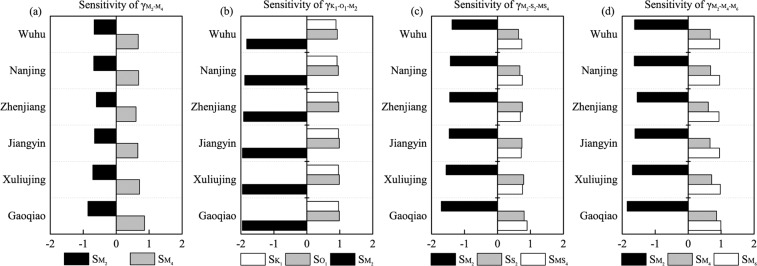
Mean value of relative sensitivity coefficients for different constituent amplitude variables contributing to tidal skewness at the six hydrological stations, generated by combinations of (**a**) M_2_/M_4_, (**b**) K_1_/O_1_/M_2_, (**c**) M_2_/S_2_/MS_4_ and (**d**) M_2_/M_4_/M_6_.

We perform a sensitivity analysis to quantify the response of the evolution of corresponding tidal amplitudes and phases to tidal skewness contributed by different combinations. The annual average relative sensitivity coefficients (RSCs) for the four major combinations (M_2_/M_4_, K_1_/O_1_/M_2_, M_2_/S_2_/MS_4_ and M_2_/M_4_/M_6_) exhibit stable spatial patterns throughout the estuary (Fig. 10). The absolute RSC values of the dominant constituent M_2_ and its first harmonic M_4_ are equal, ranging from 0.60 to 0.86 (Fig. 10a). However, the effects of their evolutions on tidal duration asymmetry are not the same with regard to the sign. The M_2_/M_4_ asymmetry is enhanced by increasing M_4_ and decreasing M_2_ amplitudes in the downstream area, while being weakened as a result of the overall tidal damping in the upstream area, which reduces nonlinear behavior. Similar characteristics have been observed elsewhere in the world, such as in the Amazon Estuary^87^, the Rhine-Meuse delta^88^ and the St. Lawrence Fluvial Estuary^28,74^.

Although astronomical tides gradually decay along the channel, their attenuation has different effects on tidal duration asymmetry. Regarding the sensitivity of the K_1_/O_1_/M_2_ triad (Fig. 10b), the RSCs for the M_2_ amplitude (${{S}}_{{M}_{2}}$) show negative values ranging from -1.82 to -1.96, suggesting that tidal skewness (${\gamma }_{{K}_{1}-{O}_{1}-{M}_{2}}$) may increase with the attenuation of the M_2_ amplitude along the channel. In contrast, the positive values of ${{S}}_{{K}_{1}}$ and ${{S}}_{{O}_{1}}$ imply that the K_1_/O_1_/M_2_ asymmetry may be reduced with a decrease in the amplitude of K_1_ and O_1_. As M_2_ damps at a faster rate than K_1_ and O_1_, the reduction in the M_2_ amplitude may result in a landward increase in the K_1_/O_1_/M_2_ asymmetry.

The sensitivity coefficients for the M_2_ amplitude (${{S}}_{{M}_{2}}$) are also negative for M_2_/S_2_/MS_4_ (Fig. 10c) and for M_2_/M_4_/M_6_ (Fig. 10d), suggesting that the decay in the M_2_ amplitude consistently enhances both the tidal duration asymmetry inherent in principal constituents and the asymmetry induced by nonlinearities in the YRE. Tidal duration asymmetry seems to be most sensitive to M_2_ amplitude variation, because the RSCs for M_2_ have the largest absolute values in each combination.

**Figure 11:**
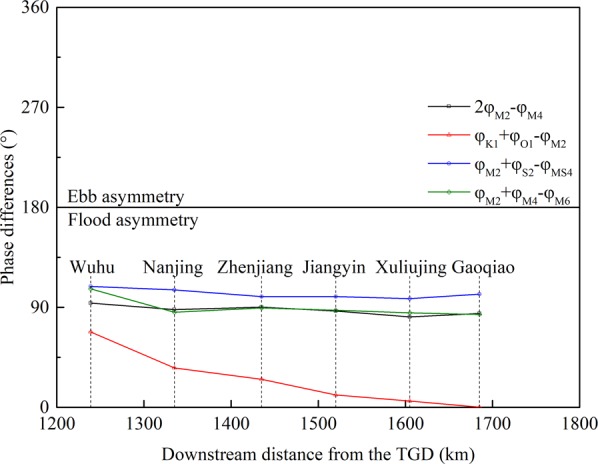
Along-channel variations in relative phase differences for four tidal combinations.

Phase differences can also play a crucial role in determining the direction of the tidal duration asymmetry^18,89^, as large-scale tidal systems usually feature obvious variations in the tidal phase differences along the channel. The relative phase differences of the four combinations ($2{\varphi }_{{M}_{2}}-{\varphi }_{{M}_{4}}$, ${\varphi }_{{K}_{1}}+{\varphi }_{{O}_{1}}-{\varphi }_{{M}_{2}}$, ${\varphi }_{{M}_{2}}+{\varphi }_{{S}_{2}}-{\varphi }_{M{S}_{4}}$ and ${\varphi }_{{M}_{2}}+{\varphi }_{{M}_{4}}-{\varphi }_{{M}_{6}}$) assessed here are all below 180°, indicating flood-dominant asymmetry in the YRE (Fig. 11). The relative phase differences of the three combinations arising from nonlinearities ($2{\varphi }_{{M}_{2}}-{\varphi }_{{M}_{4}}$, ${\varphi }_{{M}_{2}}+{\varphi }_{{S}_{2}}-{\varphi }_{M{S}_{4}}$ and ${\varphi }_{{M}_{2}}+{\varphi }_{{M}_{4}}-{\varphi }_{{M}_{6}}$) experience slight modulations along the channel, except for a relatively sharp increase in ${\varphi }_{{M}_{2}}+{\varphi }_{{M}_{4}}-{\varphi }_{{M}_{6}}$ at Wuhu. This indicates that the impact of the spatial evolution of their tidal phase differences on the corresponding tidal duration asymmetry could be limited relative to attenuating tidal amplitudes. In contrast, the relative phase difference of the astronomical constituents (${\varphi }_{{K}_{1}}+{\varphi }_{{O}_{1}}-{\varphi }_{{M}_{2}}$) significantly varies from 0° to 70° along the channel, especially in the upper reaches, in agreement with Guo *et al*.^41^. This could be another reason for the enhancement in tidal duration asymmetry of K_1_-O_1_-M_2_ toward the upstream region.

### Sensitivity of tidal duration asymmetry to river discharge

**Figure 12:**
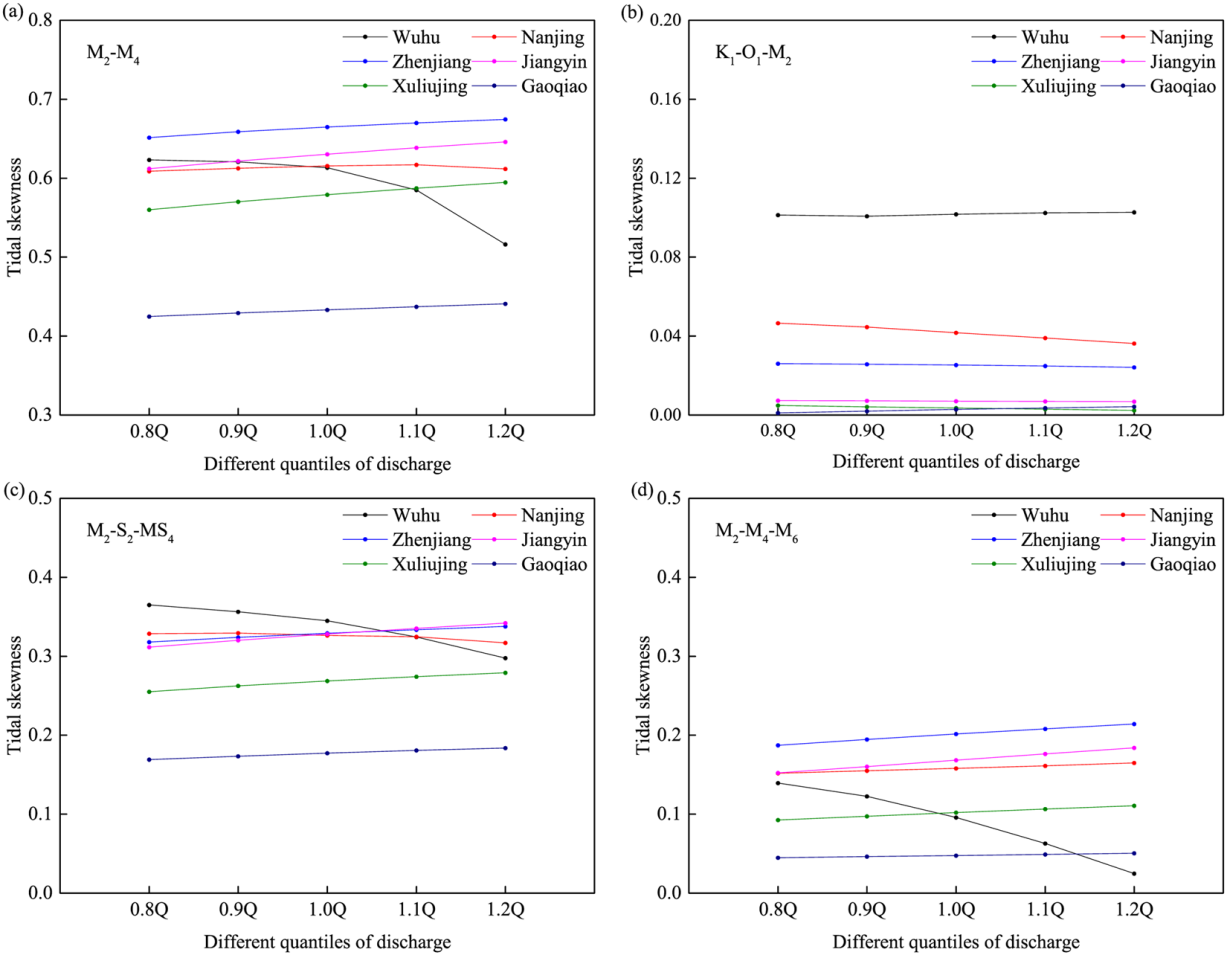
Tidal duration asymmetry for different quantiles of discharge induced by (**a**) M_2_/M_4_, (**b**) K_1_/O_1_/M_2_, (**c**) M_2_/S_2_/MS_4_ and (**d**) M_2_/M_4_/M_6_.

The tidal skewness for different quantiles of discharge shows a nonlinear response (Fig. 12) with a comparatively high sensitivity of tidal skewness to river discharge in the upper reach. At the mouth of the estuary, the tidal duration asymmetry of M_2_/M_4_ is only slightly enhanced by the increasing river discharge, but this effect becomes more significant upstream, suggesting a nonlinear transfer of energy from M_2_ to M_4_ by friction, which is enhanced upstream under higher discharge conditions. In contrast, the correlation decreases in the middle segment of the YRE and the opposite holds upstream. The flood asymmetry of M_2_/M_4_ is weakened with increasing discharge at Wuhu and Nanjing in the upper reaches. This matches the results of Guo *et al*.^23^, who use the amplitude ratio D_4_/D_2_ to explore the response of tidal duration asymmetry to increased river discharge. Compared with the asymmetry of M_2_/M_4_, the flood asymmetry generated by astronomical tides (K_1_/O_1_/M_2_) is far less sensitive to variations in discharge. In most of the estuary, only a slight reduction in the K_1_/O_1_/M_2_ asymmetry is observed with increased river discharge (Fig. 12b). Controlled by interactions between river flow and tides, the tidal duration asymmetry induced by the combination of M_2_-S_2_-MS_4_ and M_2_-M_4_-M_6_ shows a similar spatial variation to M_2_-M_4_ (Fig. 12c,d). These responses of tidal duration asymmetry to river discharge can explain the variations in tidal skewness over the pre-TGD and post-TGD periods in the wet season (Fig. 8).

### Impact of morphological changes on tidal duration asymmetry

**Figure 13:**
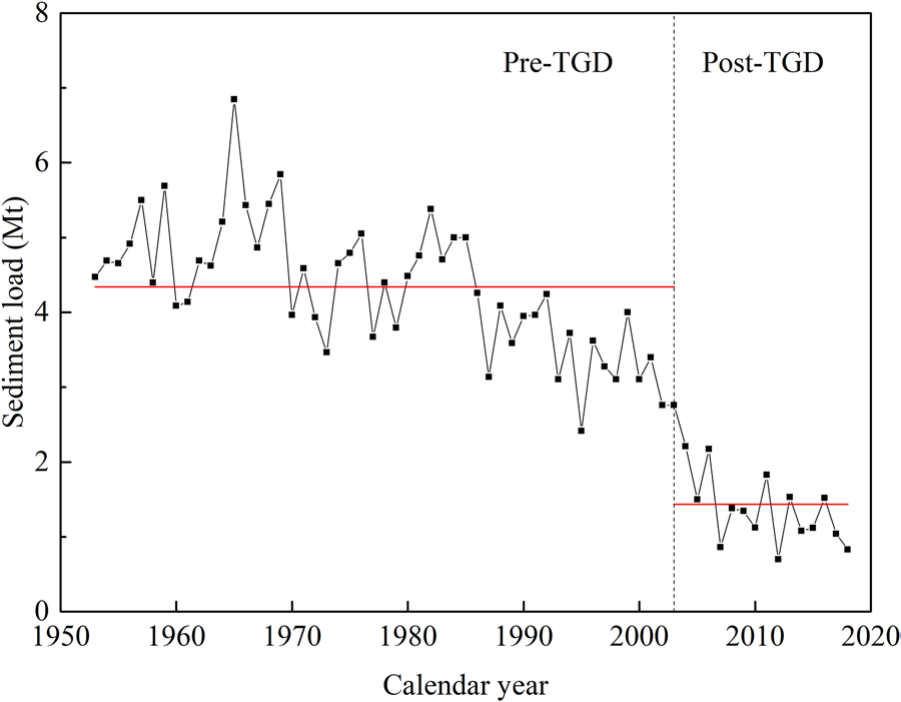
Annual sediment load for the YRE (measured at Datong). Red lines mark the average values for the pre-TGD (upper) and post-TGD (lower) periods.

The YRE has been seriously eroded since the operation of the TGD due to trapping of sediment behind the dam^27,43,90,91^, with a total erosion volume of 2.157 billion m^3^. The average annual sediment discharge measured at Datong declines by 65% (from 4.43 Mt/yr to 1.51 Mt/yr) after the TGD’s completion (Fig. 13). The TGD plays a dominant role in the reduction of sediment discharge at Datong, rather than the combined effects of other dams, precipitation and soil conservation. According to Zheng *et al*.^61^, erosion rates have varied from 0.01 to 0.19 m per year along the channel. On average, the river reach has scoured approximately ~1.2 m (from −7 m to −8.2 m) in bed elevation after the completion of the TGD. As the increase in discharge is subtle in the dry season, morphological change rather than discharge regulation could be the dominant factor altering tidal duration asymmetry.

Morphological changes play an important role in the evolution of tidal duration asymmetry. A post-TGD increase in channel depth in the YRE could lead to a decrease in flood asymmetry during the dry season (Fig. 8), along with the modulation of tidal constituents. This is consistent with previous studies on tidal duration asymmetry suggesting that shallow tidal systems tend to be more flood-dominant than deeper systems^13,14,17,20,61,92^.

## Conclusions

A nonstationary harmonic analysis method is applied to examine the spatiotemporal evolution of tidal duration asymmetry under strongly variable river discharge conditions in the YRE, divided between the pre-TGD (1965–1985) and post-TGD (2003–2014) periods. The results quantify the contributions of dual and triad tidal combinations to the overall tidal duration asymmetries, based on tidal skewness. The hydrologic and morphologic effects are assessed based on the response of tidal duration asymmetry to varying river discharge and morphological changes, with the following main findings:

(1) The tidal duration asymmetry in the YRE is flood dominant. The interaction between M_2_ and M_4_ contribute mostly to the overall tidal duration asymmetry in the entire tidal system, followed by M_2_/S_2_/MS_4_, M_2_/M_4_/M_6_, and K_1_/O_1_/M_2_. This indicates that tidal duration asymmetry within the YRE is significantly modulated by the effects of nonlinear interactions. Tidal duration asymmetry increases upstream, followed by a slight decrease, in accordance with the spatial pattern of tidal duration asymmetry induced by nonlinear effects (M_2_/M_4_, M_2_/S_2_/MS_4_ and M_2_/M_4_/M_6_), whereas tidal duration asymmetry induced by astronomical tides (K_1_/O_1_/M_2_) consistently increases upstream. These results are related to the evolution of tidal amplitudes in terms of the non-dimensional relative sensitivity coefficients. In addition, phase shifts play an important role in affecting the evolution of the K_1_/O_1_/M_2_ asymmetry in the upper reaches of the estuary, where the phase difference experiences obvious modulations.

(2) Tidal duration asymmetry varies significantly on a seasonal scale due to variations in river discharge. In the upper reaches, tidal duration asymmetry in the dry season is generally more significant than in the wet season, with the largest differences occurring furthest upstream. The opposite pattern is observed in the lower reaches, indicating that high river discharge in the wet season could accelerate energy transfer among tidal constituents. As a result, the generation of shallow-water constituents and the attenuation of astronomical constituents may lead to more asymmetric tides.

(3) The TGD plays an important role in the discharge regulation and morphological changes in the YRE. Under the hydrologic and morphologic effects, tidal duration asymmetry experiences a seasonal modulation in the YRE. Changes in river discharge are significant at a seasonal scale. The regulation of the TGD and climate change impacts in the Yangtze basin can together explain the systematic changes in seasonality. The reduced river discharge in the wet season leads to an overall increase in tidal duration asymmetry upstream, whereas the reverse occurs downstream. In addition, sediment trapping mainly caused by the TGD produces morphological changes such as rapid erosion, thus deepening channel in the YRE. These effects become dominant (relative to discharge regulation) in the dry season when the discharge regulation is slight. The deepening channel reduces the flood asymmetry in most of the YRE.

## Figures and Tables

**Figure A.1:**
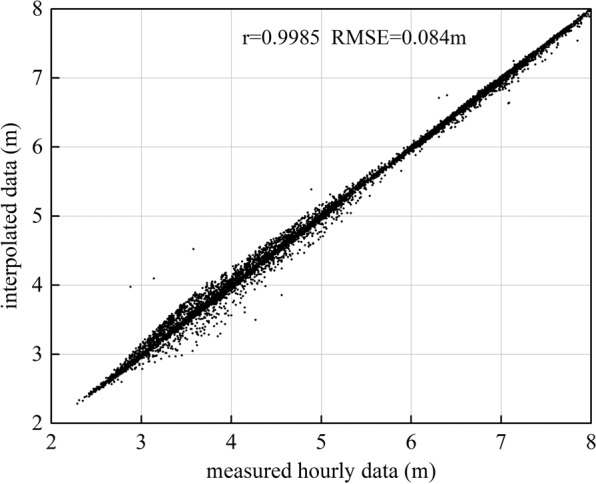
Comparison between measured hourly data and the interpolated data at Nanjing station in 2014.

**Figure A.2:**
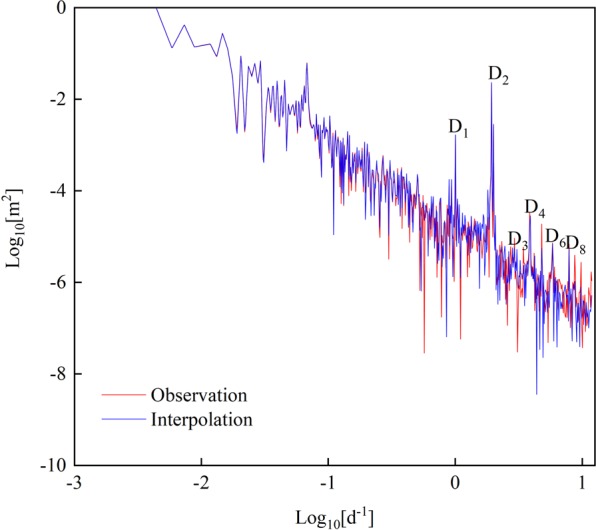
Power spectrum for interpolated (blue line) and observed (red line) water levels at Nanjing in 2014.

## Appendix A

Because of a lack of measured hourly data, trigonometrical interpolation is used to reconstruct hourly water levels based on high water-low water data. The performance of the interpolation method is here evaluated. The tidal height H(t) is modeled as:

A.1 $$H(t)=\frac{{H}_{n+1}+{H}_{n}}{2}-\frac{{H}_{n+1}-{H}_{n}}{2}\,\cos \,[\frac{\pi }{{t}_{n+1}-{t}_{n}}(t-{t}_{n})]$$

where *H*_*n*_ and *H*_*n*_ ± *1* refer to high water level and low water level respectively, *t*_*n*_ and *t*_*n*_ ± *1* are the corresponding times.

The comparison between measured hourly data and the interpolated data at Nanjing in the period of 2014 is shown in Fig. A.1. The interpolated data fits well with the measured hourly data with the Pearson correlation coefficient (r) = 0.9985. Also, the root-mean-square error (RMSE) takes a value of 0.084 m, suggesting the interpolation is reliable.

The power spectrums of observations and interpolations are compared in Fig. A.2 based on a fast Fourier Transform (FFT). It shows that the frequency structure obtained by interpolation fits the observation well at D_1_, D_2_, D_4_ and D_6_, which are mainly focused in our study. Hence, the frequency structure obtained by trigonometrical interpolation performs well for the objective of this paper. Although the higher frequency band in the spectrum (including D_8_ and higher harmonics) are not predicted well, the trigonometrical interpolation can reconstruct hourly water levels well for the purpose of our study. It’s an effective tool for the analysis of low sampling frequency water levels when high and low water data are available only.
